# Experimental Investigation on Mechanical Properties and Microstructure of Fiber-Reinforced Solid Waste-Based Foamed Lightweight Soil

**DOI:** 10.3390/ma19071436

**Published:** 2026-04-03

**Authors:** Kun Dong, Xinxin Hu, Guoxi Fan, Shutong Liu, Zhuo Liu

**Affiliations:** Engineering College, Ocean University of China, Qingdao 266100, China; dongkun@ouc.edu.cn (K.D.); huxx0720@163.com (X.H.); liushutong@ouc.edu.cn (S.L.); lz9797@stu.ouc.edu.cn (Z.L.)

**Keywords:** polyvinyl alcohol fibers, red mud, solid waste, foamed lightweight soil, mechanical property

## Abstract

Conventional cement-based foamed lightweight soil (FLS) faces cost and environmental challenges. This study develops a sustainable polyvinyl alcohol (PVA) fiber-reinforced solid waste-based FLS (PVA-SWFLS) by entirely replacing cement with a ternary system of red mud, granulated blast furnace slag, and fly ash. PVA fibers were incorporated to mitigate inherent brittleness and cracking. The effects of fiber content (0–0.9 vol%), length (3–15 mm), water–binder ratio (0.35–0.55), and wet density (550–950 kg/m^3^) on the fluidity and compressive strength were evaluated, along with analyses of microstructure and pore characteristics using scanning electron microscopy and mercury intrusion porosimetry. Findings reveal that fiber addition reduces flowability (up to 34.9%) but significantly bolsters compressive strength, depending on fiber content and length. For 0.3% and 0.5% contents, optimal fiber lengths of 12 mm and 9 mm were observed, respectively; the 28-day compressive strength reached a maximum of 2.97 MPa at the 0.3% content with 12 mm fibers. Beyond these optimal points, and particularly for higher contents (0.7–0.9%), strength decreased monotonically with increasing fiber length due to fiber agglomeration and reduced compactness. Furthermore, strength correlated positively with wet density and negatively with the water–binder ratio, while fluidity increased with both. The hierarchy of influence was identified as: fiber content > fiber length, and wet density > water–binder ratio, while all four parameters significantly governed fluidity. The stress–strain behavior under different parameter combinations was analyzed, and a parametric constitutive model was established to support practical applications.

## 1. Introduction

The construction industry is a major contributor to global carbon emissions [1], while the disposal of industrial solid wastes such as red mud poses serious environmental challenges [2]. Developing sustainable construction materials that utilize industrial by-products to replace cement has therefore become a critical research priority. Foamed lightweight soil (FLS)—a material widely used in applications such as road subgrades and backfilling due to its lightweight nature and thermal insulation—is typically manufactured with cement as the primary binder. However, its conventional production relies heavily on cement, which entails high costs, resource depletion, and environmental pollution—limitations that motivate the search for alternative cementitious materials [3].

Red mud (RM) is a highly alkaline by-product of alumina refining, with global annual output exceeding 175 million tons and total stockpiles reaching approximately 4 billion tons—yet less than 5% is currently utilized [4,5]. Rich in active components such as alumina, calcium oxide, and silica, RM offers potential as a cement substitute that has motivated extensive research [6,7,8]. Studies indicate that RM can enhance early-age strength by accelerating clinker hydration [9,10]. However, this enhancement is offset by strength loss at higher replacement levels, which restricts its practical application in mortar [11].

To further expand the utilization of RM, increasing attention has been paid to composite cementitious systems that combine RM with other industrial by-products [12]. Ground granulated blast furnace slag (GGBS) is widely recognized as an effective cement substitute due to its latent hydraulic reactivity [13,14], and numerous studies have demonstrated its excellent performance after alkali activation [15]. For instance, Li et al. [16] demonstrated that incorporating GGBS into RM-based systems enhances compressive strength through the formation of C-A-S-H gels and microstructural densification, while Wang et al. [17] reported a synergistic effect where 10% RM compensated for strength loss and improved early-age hydration. These findings confirm that GGBS can effectively activate RM and improve mechanical performance. Fly ash (FA), rich in reactive SiO_2_ and Al_2_O_3_, has also been explored as a substitute for clay minerals in cement production [18]. Wang et al. [19] reported that an optimal RM/FA ratio of 6:4 maximized compressive strength (2.70 MPa) due to RM’s alkaline activation of FA’s reactive components, promoting C-A-S-H and N-A-S-H gel formation. However, the strength reduction upon mixing FA and RM alone underscores the need for additional components like GGBS to stabilize the system [20]. Consequently, the synergistic use of RM, GGBS, and FA—as demonstrated in ternary systems [21]—represents a viable strategy for developing alternative cementitious materials while promoting solid waste valorization.

The utilization of FLS has become widespread, and RM has also been employed in its preparation to further reduce material costs. Studies have shown that moderate RM content (e.g., 20%) can be beneficial: Ou et al. [22] reported comparable compressive strength to RM-free specimens due to secondary hydration, while Xiong et al. [23] observed pore refinement in alkali-activated foamed systems with 20% RM, with the proportion of pores smaller than 500 μm increasing from 81.7% to 85.8%. However, higher RM contents lead to agglomeration, pore deterioration, and strength loss [24,25]. Despite these findings, RM is currently used primarily as a low-volume admixture in FLS production, and the application of red mud-based composite cementitious systems in FLS remains limited, an issue that the present study aims to address.

FLS is inherently brittle and prone to cracking under external loading, which limits its practical applications [26]. Fiber reinforcement is an effective strategy to mitigate this drawback. A three-dimensional fiber network can bridge cracks, redistribute stress, and improve ductility [27,28,29], enhancing mechanical properties such as compressive, flexural, and tensile strengths [30,31,32]. Among various fiber types, synthetic fibers—particularly polyvinyl alcohol (PVA) fibers—have attracted attention due to their excellent bonding with cementitious matrices [33]. Studies have shown that PVA fibers can increase the compressive and flexural strength of foam concrete by up to 47.4% and 190.3%, respectively, while refining pore structure and improving fiber distribution [34]. Given these advantages—particularly the proven interfacial bonding in alkali-activated systems and substantial improvements in mechanical properties—PVA fibers are selected as the reinforcement material in the present study.

In summary, most existing studies on red mud utilization in cementitious materials have focused on partial replacement of cement, with replacement rates typically limited to 10–30% to avoid significant strength loss. In contrast, this study aims to achieve 100% cement replacement in FLS by developing a solid waste-based binder system composed entirely of RM, GGBS, and FA. However, such a solid waste-based FLS—while maximizing the utilization of industrial by-products—inevitably exhibits lower mechanical strength, which limits its practical application. To overcome this limitation, PVA fibers are incorporated to reinforce the matrix. Accordingly, this study develops a PVA fiber-reinforced solid waste-based foamed lightweight soil (PVA-SWFLS) using RM, GGBS, and FA as the sole cementitious materials. The experimental program was designed to systematically investigate: (i) the effects of fiber length (3–15 mm) and fiber content (0–0.9 vol%), as well as water–binder ratio (0.35–0.55) and wet density (550–950 kg/m^3^), on compressive strength and fluidity of this solid waste-based FLS, including their interactive effects and optimal parameter combinations; (ii) the microstructural characteristics and pore structure features of PVA-SWFLS using scanning electron microscopy (SEM) and mercury intrusion porosimetry (MIP) to elucidate the fiber reinforcement mechanisms; and (iii) the stress–strain behavior under varying mix design parameters, leading to a parametric constitutive model that accounts for the influence of water–binder ratio and wet density for practical engineering applications. The findings are expected to provide a better understanding of fiber reinforcement mechanisms in solid waste-based foamed lightweight soil and offer practical guidance for optimizing mix designs toward large-scale utilization of red mud and other industrial by-products in sustainable construction.

## 2. Materials and Test Methods

### 2.1. Materials

**Figure 1:**
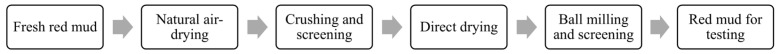
Preparation process of red mud powder.

The Bayer red mud used in the test was procured from the Shandong Liaocheng Xinfa Group (Liaocheng, China). After natural air-drying, the larger red mud blocks were crushed and then dried in an electrothermal blowing dry box (101-3B, Shaoxing Supo Instrument Co., Ltd., Shaoxing, China) at 105 °C for 24 h. The dried red mud was then ground in a planetary ball mill and sieved through a 325-mesh screen to obtain the desired red mud powder. The preparation process is illustrated in Figure 1.

**Figure 2:**
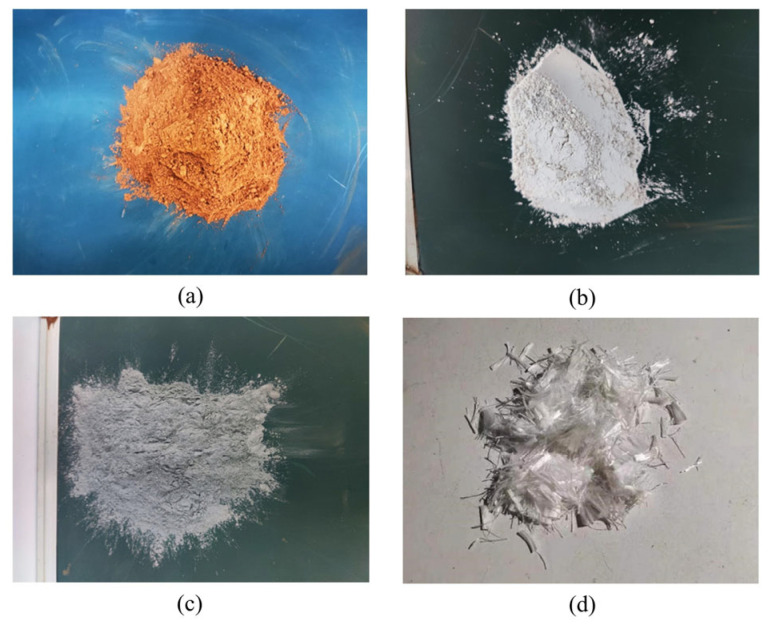
Test raw materials: (**a**) Red mud powder after grinding; (**b**) GGBS; (**c**) FA; (**d**) PVA.

**Figure 3:**
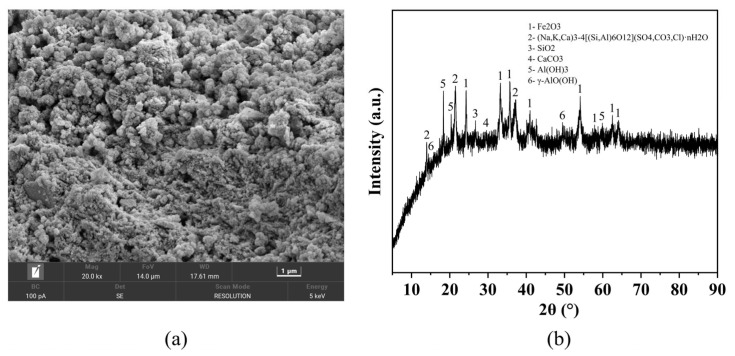
Detection image of RM: (**a**) SEM image (20.0 k magnification); (**b**) XRD pattern.

Figure 2 shows the raw materials used in this study. The SEM image of RM is shown in Figure 3a, revealing a porous, irregularly shaped, and unevenly sized granular microstructure. The X-ray diffraction (XRD) pattern was obtained using a Rigaku Miniflex 600 diffractometer (Rigaku Corporation, Tokyo, Japan) with Cu Kα radiation, scanning over a 2θ range of 5–90° at a speed of 5°/min. The results are presented in Figure 3b. The main mineralogical compositions of RM are as follows: hematite (Fe_2_O_3_), cancrinite ((Na, K, Ca)_3_-4[(Si, Al)_6_O_12_](SO_4_, CO_3_, Cl)·nH_2_O), quartz (SiO_2_), calcite (CaCO_3_), gibbsite (Al(OH)_3_) and boehmite (γ-AlO(OH)).

The chemical compositions of RM, GGBS, and FA were determined by X-ray fluorescence (XRF) spectrometry using a Panalytical Zetium spectrometer (Malvern Panalytical B.V., Almelo, The Netherlands), and the results are presented in Table 1. From a reactivity perspective, the high Fe_2_O_3_ content in RM (46.1%) contributes to its dense microstructure, while the presence of reactive SiO_2_ and Al_2_O_3_ (13.9% and 22.2%, respectively) serves as the main source of reactive components for alkali-activated reactions. The Na_2_O content (10.3%) may contribute to the alkaline environment, partially reducing the demand for external alkali activators. The grinding and sieving process (325-mesh, particle size ≤ 45 μm) produced red mud powder with a specific surface area of approximately 70–100 m^2^/g. This high specific surface area, combined with the porous and irregular morphology observed in SEM, provides abundant contact points between RM particles and the alkali activator, facilitating the formation of hydration products and enhancing the alkali activation reactivity.

S95 grade GGBS with a density of 2.83 g/cm^3^ and a specific surface area of 400 m^2^/kg, and low-calcium FA were used in the study. The composite foaming agent was selected for the test, and the relevant properties are shown in Table 2. The foaming agent was diluted at a rate of 1:40 and formed a foam through a foaming machine. Sodium silicate solution (water glass) with an initial modulus of 3.22 (SiO_2_ = 26.5%, Na_2_O = 8.5%) was used as the alkali activator. NaOH granules (purity ≥ 99%) were added to adjust the modulus to 2.0. The activator dosage was controlled based on its Na_2_O content: the amount added to each mixture was calculated to provide an Na_2_O mass equivalent to 6% of the total binder mass (RM, GGBS, and FA combined). The physical properties of the PVA fibers, including five different lengths (3, 6, 9, 12, and 15 mm), are listed in Table 3. The standard sand used in this study was procured from a company in Xiamen, China, while the test mixing water was from Qingdao Waterworks. In this study, RM, GGBS, and FA collectively serve as the sole binder system for the subsequent preparation of foamed lightweight soil. No additional soil or filler material is introduced; the binder itself forms the matrix of the lightweight soil.

### 2.2. Test Methods

(1) Test methods for solid waste binder system

**Figure 4:**
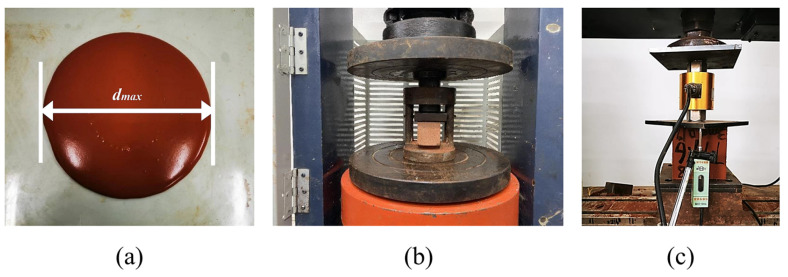
Test methods for solid waste binder system and FLS: (**a**) Flowability test; (**b**) compressive strength test for binder system; (**c**) compressive strength test for FLS.

The fluidity of the solid waste cementitious materials was tested according to the Chinese standard GB/T8077-2012 [35], as illustrated in Figure 4a. The water–binder ratio was fixed at 0.5 for all binder mixtures. The compressive strength of the binder system was determined following the Chinese standard GB/T17671-2021 (ISO method) [36]. In accordance with this method, prism specimens (40 mm × 40 mm × 160 mm) were first subjected to a flexural strength test, followed by the compressive strength test (as shown in Figure 4b). This study focuses on the compressive strength results. Specimens were cured under standard conditions, and compressive strength was measured at 3, 7, and 28 days using a compression testing machine (model YAW-2000B, Jinan Kuangyan Testing Instrument Co., Ltd., Jinan, China). For each mix, three specimens were tested at each age, and the average value was reported.

(2) Test methods for FLS

The fluidity of the fresh mixture was tested in accordance with Appendix D of the Chinese standard CJJ/T177-2012 [37], as illustrated in Figure 4a. The fluidity was maintained within 160–200 mm, as recommended by the standard. The wet density of the fresh mixture was measured according to Appendix C, and was required to be within γ ± 50 kg/m^3^, where γ is the target design density. The mechanical properties of the specimens were characterized by unconfined cubic compressive strength, as determined in accordance with the Chinese standard GB/T11969-2008 [38] as shown in Figure 4c. The compressive test was conducted using a universal testing machine (model WDW-100, Changchun Kexin Testing Instrument Co., Ltd., Changchun, China), and a displacement meter was configured to obtain the load–displacement curve during the experiment. Three specimens were tested for each condition, and the average value was taken as the final result for compressive strength at 7 d and 28 d.

**Figure 5:**
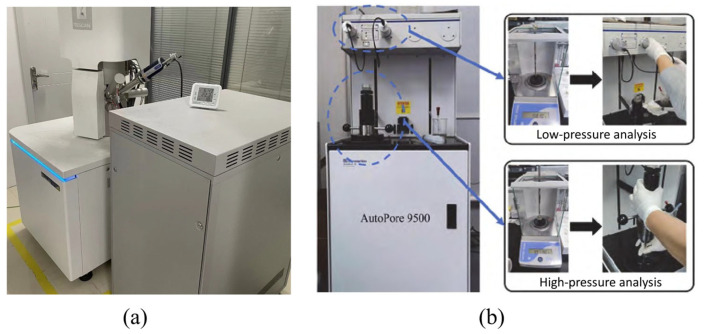
Testing equipment: (**a**) SEM (TESCAN MIRA LMS); (**b**) MIP (Micromeritics AutoPore IV 9500).

To analyze the microstructure of the PVA-SWFLS, specimens with different fiber parameters (Groups P) and different mix proportions (Groups W) were examined using a TESCAN MIRA LMS scanning electron microscope (TESCAN, Brno, Czech Republic), as illustrated in Figure 5a. Due to the non-conductive nature of the samples, gold sputter coating was applied before observation. To analyze the pore structure, dried specimens from varying wet densities and water–binder ratios (Group W) were subjected to MIP using a Micromeritics AutoPore IV 9500 instrument (Micromeritics Instrument Corporation, Norcross, GA, USA), as shown in Figure 5b.

## 3. Optimization of Solid Waste Binder System

To effectively utilize these solid waste binders and maximize RM consumption, a preliminary study was conducted to optimize the proportions of RM, GGBS, and FA. This provides a theoretical basis for the subsequent step in the preparation of the reinforced foamed lightweight soil and the design of the ratio.

### 3.1. Experimental

**Figure 6:**
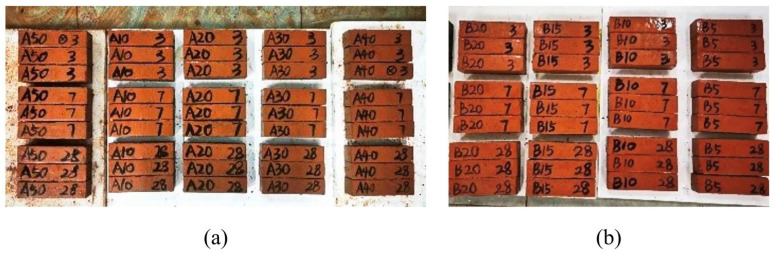
Specimens with different mixing ratios: (**a**) Group A; (**b**) Group B.

In this study, RM, GGBS, and FA are collectively used as the binder system for alkali-activated solid waste cementitious materials. To maximize RM utilization while ensuring adequate mechanical performance, a preliminary optimization of their proportions was conducted as follows. The test utilized RM, GGBS, and FA as solid waste precursors, together with sodium silicate solution (modulus 2.0, dosage 6%) as an alkali activator to stimulate their latent hydraulic and pozzolanic reactivity, initiating the formation of binding gels. Two series of specimens were prepared: RM-GGBS blends (Group A) and RM-GGBS-FA blends (Group B). The mass ratio of cementitious material to standard sand was 1:3, and the water–binder ratio was 0.5. The mix proportions are given in Table 4. For each mix, nine prism specimens (40 mm × 40 mm × 160 mm) were cast. Figure 6 shows specimens with different mixing ratios. All specimens were prepared and tested following the methods described in Section 2.2.

### 3.2. Results

In accordance with the aforementioned mix proportion scheme, specimens from each group were subjected to testing.

(1) Group A

**Figure 7:**
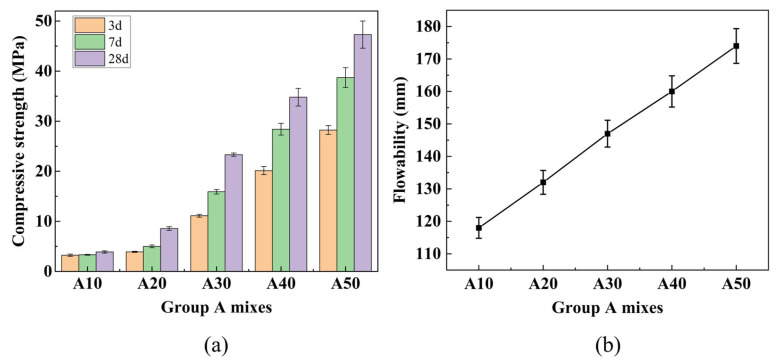
Results for Group A mixes: (**a**) Compressive strength at different ages; (**b**) fluidity.

RM contains calcium, silicon, and aluminum compounds, but their low concentrations and limited reactivity result in poor mechanical performance when RM content is high. Therefore, incorporating active materials such as GGBS is necessary. Figure 7a shows the compressive strength development of Group A mixes at different ages. Under standard curing conditions, the 28-day compressive strength increased steadily with GGBS content, reaching 34.79 MPa at 40% GGBS and 47.29 MPa at 50% GGBS. These values satisfy the strength requirements for P.O 32.5 cement (≥32.5 MPa) and P.O 42.5 cement (≥42.5 MPa), respectively. The comparison with conventional cement strength grades is intended only as a reference for material development, considering the differences in test methods (mortar prisms vs. concrete cubes) and hydration mechanisms.

Figure 7b shows the fluidity results of Group A mixes. As the GGBS content increased from 10% to 50% (corresponding to RM:GGBS ratios from 9:1 to 5:5), the flowability increased progressively from 118 mm to 174 mm, representing a 47% improvement. This increase is attributed to the more regular shape and uniform particle size distribution of GGBS particles compared to RM, which reduces interparticle friction and enhances lubrication [39]. In contrast, RM particles are irregular and tend to agglomerate at high contents, increasing friction and reducing flowability [40].

Based on the compressive strength and fluidity results, a GGBS content of 40% was selected to balance RM utilization and mechanical performance.

(2) Group B

**Figure 8:**
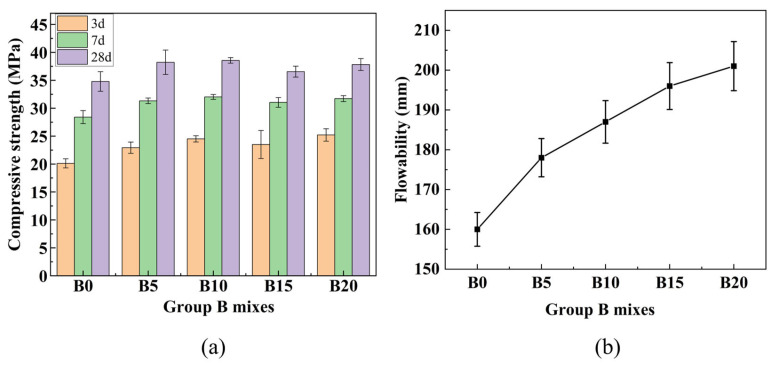
Results for Group B mixes: (**a**) Compressive strength at different ages; (**b**) fluidity.

As illustrated in Figure 8a, the compressive strength of Group B specimens first increased and then slightly decreased with increasing FA content. The maximum 28-day strength reached 38.55 MPa at 10% FA content, representing a 10.8% increase compared to the FA-free mix B0 (which has the same composition as A40: 60% RM + 40% GGBS). This enhancement can be attributed to the synergistic effect of FA: its finer particles fill voids, improving packing density, while its additional reactive silica and alumina contribute to the formation of hydration products. Beyond 10% FA, excessive dilution of the binder may offset these benefits, leading to a slight strength reduction.

Figure 8b presents the fluidity results of Group B mixes with varying FA content. As the FA content increased from 0% to 20%, the flowability rose from 160 mm to 201 mm, with increments of 178 mm at 5% FA, 187 mm at 10% FA, and 196 mm at 15% FA. The increase can be attributed to the spherical morphology of FA particles, which act as lubricants and reduce interparticle friction [39]. Additionally, FA increases the total surface area, improving water distribution. Although flowability continued to increase up to 20% FA, the rate of increase gradually slowed, possibly due to increased water adsorption on the larger surface area of FA, which reduces free water content.

Based on the experimental results, the optimal binder composition of 50% RM, 40% GGBS, and 10% FA was selected for subsequent preparation of PVA-reinforced solid waste-based foamed lightweight soil.

## 4. Experimental Design for SWFLS

### 4.1. Specimen Design

Based on the optimized binder composition (RM:GGBS:FA = 5:4:1) obtained from the preliminary study, PVA-SWFLS mixtures were designed with varying fiber parameters and mix proportions. The binder system itself constitutes the matrix of the foamed lightweight soil, and no additional soil or aggregate is introduced. To investigate the effects of fiber length and content, the design wet density was set at 750 kg/m^3^ and the water–binder ratio at 0.45 according to the results of the preliminary experiment.

Five distinct fiber lengths (3 mm, 6 mm, 9 mm, 12 mm, and 15 mm) and five volumetric contents (0.1%, 0.3%, 0.5%, 0.7%, and 0.9%) were selected, while a fiber-free mixture with the same design wet density (750 kg/m^3^) and water–binder ratio (0.45) was used as a control group (CG). Subsequently, with fiber length fixed at 12 mm and content at 0.3%, three wet densities (550 kg/m^3^, 750 kg/m^3^, and 950 kg/m^3^) and three water–binder ratios (0.35, 0.45, and 0.55) were investigated. The experimental mix proportion design is shown in Table 5.

Due to the sequential experimental design, interaction effects between fiber parameters and mix proportions were not investigated in this study.

### 4.2. Preparation and Maintenance of Samples

**Figure 9:**
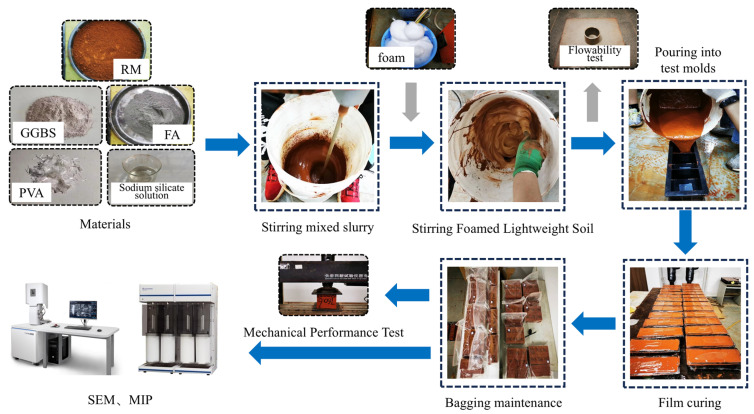
Sample preparation and test process.

Figure 9 shows the sample preparation and testing process. First, the weighed raw materials (RM, FA, and GGBS) were poured into the mixer and mixed to form a homogeneous dry mixture. Subsequently, the weighed sodium silicate solution, pre-saturated PVA fibers (prepared by soaking in water for 24 h and then draining), and water were added to the mixture and mixed until a uniform slurry with well-dispersed fibers was obtained. Secondly, the diluted blowing agent was used to prepare foam through the blowing machine. Finally, a measured quantity of foam was incorporated into the slurry and mixed for 2 min at a controlled speed of 200 r/min to achieve uniform distribution while avoiding foam collapse. The stirred slurry was then cast into 100 mm × 100 mm × 100 mm molds and covered with plastic film to prevent moisture loss. The specimens were stored in a constant temperature and humidity curing cabinet for 72 h prior to demolding. After demolding, the specimens were placed in sealed plastic bags and maintained at 95% ± 3% relative humidity and 20 ± 2 °C for 7 d and 28 d, respectively, before testing.

## 5. Results and Discussion

### 5.1. Fluidity

**Figure 10:**
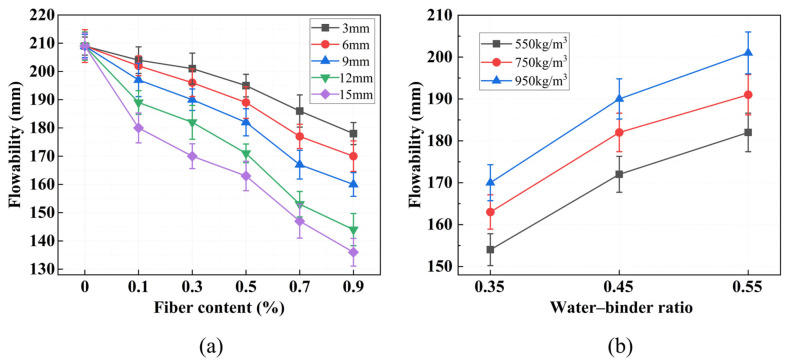
Flowability test results: (**a**) Flowability at different fiber lengths and contents; (**b**) flowability at different wet densities and water–binder ratios.

The results of the mixture flowability test are shown in Figure 10. As demonstrated in Figure 10a, the impact of varying fiber lengths and fiber contents on the flowability of the FLS is pronounced. In the absence of fiber content, the flowability of the FLS is measured at 209 mm. However, as the length of fiber and fiber content are increased, the flowability undergoes a gradual decrease. When the fiber length is 15 mm and the volume content is 0.9%, the fluidity of the FLS is only 136 mm, which is a reduction of 34.9% compared to the CG. This indicates that fiber addition significantly impairs the workability of the fresh mixture, with the effect being more pronounced at higher fiber contents and longer fiber lengths. This phenomenon can be attributed to the interaction between the PVA fiber and the lightweight soil slurry. The presence of PVA fibers results in the formation of an adsorption layer on the fiber–matrix interface, which increases the internal friction between solid particles and restricts the flow of the fresh mixture [33]. Studies have shown that fibers increase the yield stress and plastic viscosity of the slurry, leading to reduced workability [41,42]. The effect becomes more pronounced at higher fiber contents due to increased fiber–fiber interactions and mechanical interlocking.

As shown in Figure 10b, under constant fiber conditions, fluidity increases with both water–binder ratio and design wet density. The increase in fluidity with higher water–binder ratio can be explained by the lubricating effect of excess free water, which reduces interparticle friction [43]. The positive correlation between wet density and fluidity, while seemingly counterintuitive, can be explained from a rheological perspective. At higher wet densities, the foam content decreases, which reduces the volume fraction of the viscous foam phase that hinders flow. This leads to a lower plastic viscosity of the fresh mixture, thereby improving its flowability [33]. Additionally, with reduced foam content, the cementitious paste becomes more continuous and homogeneous, decreasing the internal friction between solid particles and further enhancing fluidity. Studies on foam concrete rheology have shown that mixtures with lower foam volumes exhibit improved workability due to reduced viscosity and enhanced slurry stability, which is consistent with the present findings [43].

### 5.2. Failure Mode

**Figure 11:**
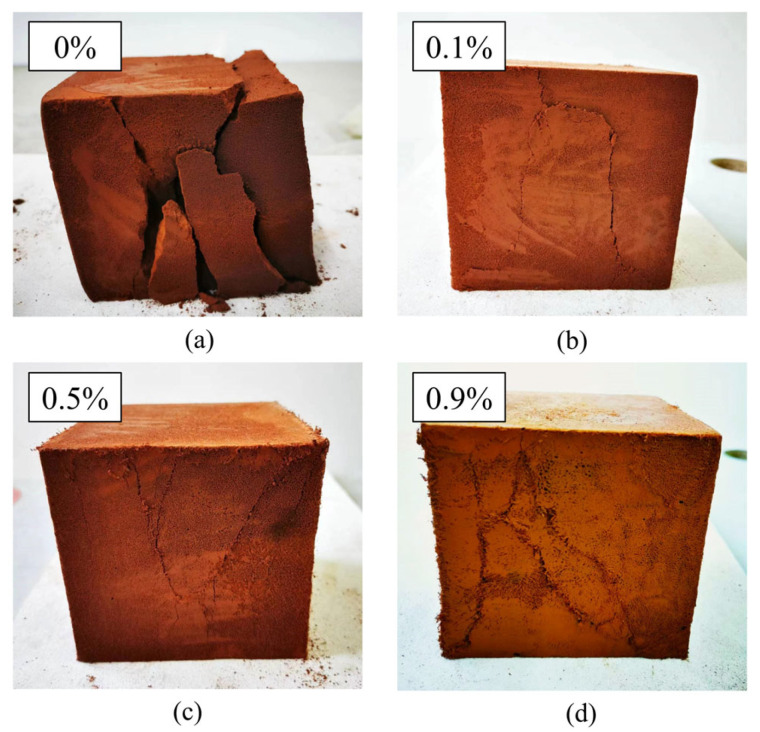
Compressive failure modes of typical specimens with different fiber contents: (**a**) Without fiber; (**b**) fiber content of 0.1%; (**c**) fiber content of 0.5%; (**d**) fiber content of 0.9%.

Figure 11 shows the compression failure modes of typical specimens with different fiber contents. In the CG without fibers, vertical micro-cracks initiated at internal voids under compressive loading. As the applied load increased, the cracks propagated and gradually coalesced into continuous splitting planes, ultimately leading to vertical splitting failure with pronounced block spalling, as illustrated in Figure 11a. With increasing fiber content, the failure mode exhibited a progressive transition in crack orientation, while crack width followed a non-monotonic trend. At 0.1% fiber content, vertical cracks remained dominant but showed reduced width compared to the CG. At 0.5% fiber content, a mixed failure mode was observed, with both vertical splitting and inclined shear cracks at approximately 45°; among the specimens shown in Figure 11, cracks were finest at 0.5% fiber content. At 0.9% fiber content, the failure was predominantly characterized by inclined shear cracking, with only minor vertical cracking. However, cracks at 0.9% were more pronounced than at lower fiber contents, suggesting that excessive fiber addition may introduce defects that facilitate crack propagation, as shown in Figure 11b–d.

The analysis demonstrates that the FLS predominantly experiences compressive stresses, whereas the PVA fibers primarily bear the transverse tensile stresses that arise during cracking due to compressive deformation. The incorporation of fibers resulted in the specimen no longer exhibiting vertical cracks during failure. Concurrently, the crack exhibited a gradual shift from the overall split state of the specimen to the edge portions, which also led to a gradual enhancement in the specimen’s integrity. Furthermore, the integration of the fiber ensures that any FLS fragments that may have detached remain connected to the primary structure, thereby preserving its integrity.

This transition from vertical splitting failure to oblique cracking failure indicates that fiber incorporation significantly enhanced the ductility and toughness of the FLS. The fibers effectively bridged cracks and redistributed stresses through fiber–matrix interfacial bonding and mechanical interlocking [33], enabling the material to undergo greater deformation before failure and maintain integrity after cracking—a critical improvement for engineering applications where post-peak behavior is important.

The compressive damage patterns of foamed lightweight soil specimens with different wet densities and water–binder ratios were similar to those described above, and are therefore not discussed separately.

### 5.3. Stress–Strain Curve

The uniaxial compressive test was conducted on the foamed lightweight soil with an age of 28 days. The stress–strain curves were obtained by converting the load–displacement data from the universal testing machine using the standard relationships: stress σ=N/A and strain ε=Δl/l, where N is the axial load (N), A is the cross-sectional area (mm^2^), Δl is the compressive deformation (mm), and l is the original height of the specimen (mm).

(1) Group P

**Figure 12:**
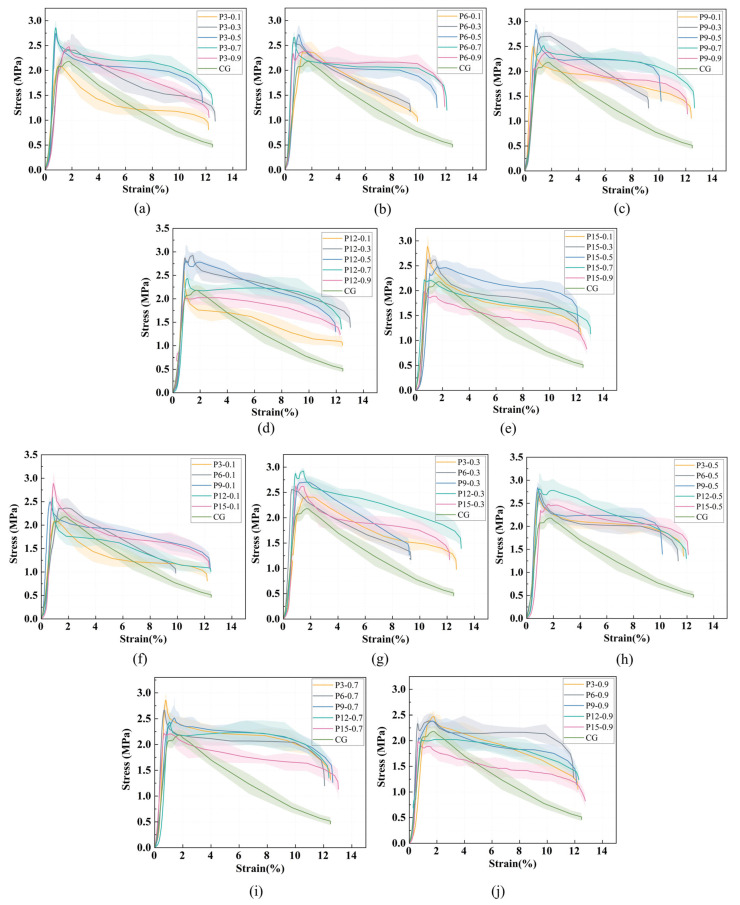
Stress–strain curves of foamed lightweight soil with different fiber content (length) at the same fiber length (content): (**a**–**e**) show fiber lengths of 3, 6, 9, 12, and 15 mm; (**f**–**j**) show fiber contents of 0.1%, 0.3%, 0.5%, 0.7%, and 0.9%.

Figure 12a–e shows the stress–strain curves of foamed lightweight soil with varying fiber content at constant fiber length, while Figure 12f–j show the curves with varying fiber length at constant fiber content. The vertical drop in the descending branch is due to the test setup where loading was terminated when a preset displacement was reached.

As shown in Figure 12, all stress–strain curves exhibit a similar three-stage morphology: elastic stage, plastic yielding stage, and post-peak failure stage. In the elastic stage, stress increases linearly with strain, with the load primarily borne by the FLS matrix. In the plastic yielding stage, microcracks develop and propagate, and the PVA fibers begin to share the load by resisting transverse tensile stresses. In the post-peak stage, strain softening occurs, with stress decreasing rapidly at first and then more gradually.

The stress–strain behavior varied quantitatively with fiber parameters. The CG (control group without fibers) exhibited a brittle failure characterized by a sharp post-peak stress drop, with a peak stress of 2.18 MPa and a peak strain of 1.77%. With fiber addition, both peak stress and peak strain changed significantly across the tested range. Across all fiber-reinforced specimens, the peak stress ranged from 1.98 MPa to 2.87 MPa. A consistent non-monotonic trend was observed with respect to both fiber content and fiber length. For a given fiber length, the peak stress initially increased with fiber content, reached a maximum at an optimal content (typically 0.3% or 0.5%), and then decreased at higher contents. Similarly, for a given fiber content, the peak stress increased with fiber length up to an optimal length (typically 9–12 mm) and then declined at 15 mm. The peak strain across all fiber-reinforced specimens ranged from 0.60% to 1.89%, showing no clear correlation with either fiber content or length.

The highest peak stress (2.87 MPa) was achieved at a fiber length of 12 mm and a fiber content of 0.3%, representing a 31.7% increase compared to the CG. This combination also exhibited the most gradual post-peak stress decline, indicating enhanced ductility. Some specimens showed a slight stress increase after the initial post-peak drop, which can be attributed to fiber bridging and pull-out effects. This behavior confirms that fiber reinforcement improves both strength and toughness of the foamed lightweight soil.

(2) Group W

**Figure 13:**
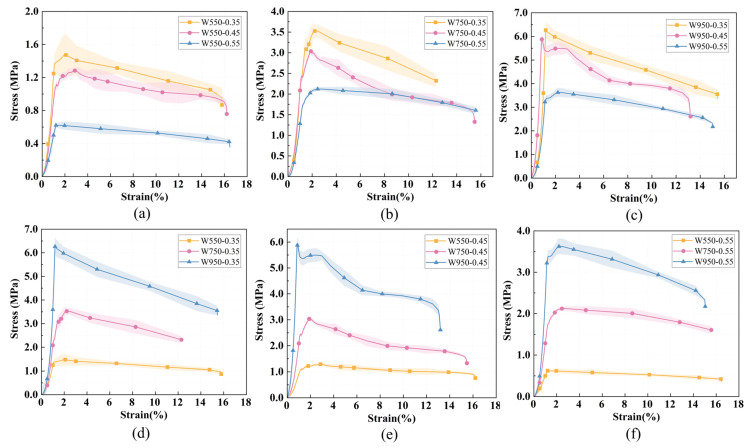
Stress–strain curves of foamed lightweight soil with different water–binder ratio (wet density) at the same wet density (water–binder ratio): (**a**–**c**) show wet densities of 550, 750, and 950 kg/m^3^; (**d**–**f**) show water–binder ratios of 0.35, 0.45, and 0.55.

Figure 13a–c show the stress–strain curves for different water–binder ratios at constant wet density, while Figure 13d–f show the curves for different wet densities at a constant water–binder ratio. The vertical drop in the descending branch is due to the test setup where loading was terminated when a preset displacement was reached.

As shown in Figure 13, all stress–strain curves exhibit the same three-stage morphology observed in Group P: elastic stage, plastic yielding stage, and post-peak failure stage. In the elastic stage, stress increases linearly with strain. In the plastic yielding stage, microcracks develop and propagate. In the post-peak stage, strain softening occurs, with stress decreasing gradually after reaching its peak.

The stress–strain behavior varied quantitatively with mix parameters. For all Group W specimens, the peak stress ranged from 0.62 MPa to 6.26 MPa. At a constant wet density, increasing the water–binder ratio consistently reduced the peak stress. For example, at a wet density of 750 kg/m^3^, the peak stress decreased from 3.52 MPa at a water–binder ratio of 0.35 to 3.03 MPa at 0.45, and further to 2.12 MPa at 0.55; similar trends were observed at wet densities of 550 kg/m^3^ and 950 kg/m^3^. Conversely, at a constant water–binder ratio, increasing the wet density substantially increased the peak stress. At a water–binder ratio of 0.45, the peak stress rose from 1.28 MPa at 550 kg/m^3^ to 3.03 MPa at 750 kg/m^3^, and reached 5.88 MPa at 950 kg/m^3^; this trend was consistent across all tested water–binder ratios.

The peak strain across all Group W specimens ranged from 0.88% to 2.93%, showing no consistent correlation with either water–binder ratio or wet density. This indicates that while mix parameters significantly affect stress magnitude, they have limited influence on the deformation capacity at peak load. The highest peak stress (6.26 MPa) was achieved at a wet density of 950 kg/m^3^ and a water–binder ratio of 0.35, representing the optimal combination for maximizing compressive strength within the tested range.

### 5.4. Compressive Strength

(1) Group P

**Figure 14:**
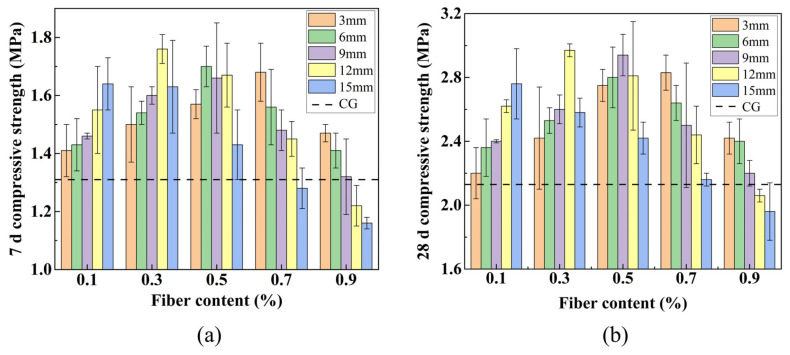
Compressive strength at different fiber lengths and contents: (**a**) 7 d; (**b**) 28 d.

Figure 14 illustrates that the compressive strength of FLS is influenced by variations in fiber lengths and contents. As demonstrated in Figure 14, the 7 d and 28 d compressive strengths of most fiber-reinforced specimens were higher than those of the control group, with the exception of P12-0.9, P15-0.7, and P15-0.9. The 28-day compressive strength of the CG was 2.13 MPa. The 28-day compressive strength reached a maximum of 2.97 MPa at a fiber length of 12 mm and a fiber content of 0.3%, representing increases of 34.4% and 39.4% at 7 d and 28 d, respectively, compared to the CG. This confirms that fiber incorporation substantially enhances the compressive properties of FLS.

It can also be observed that when the fiber length is held constant, the compressive strength of specimens measuring 3 mm, 6 mm, 9 mm, and 12 mm demonstrates a tendency to increase and then decrease with the increase in fiber content. Conversely, the compressive strength of specimens measuring 15 mm demonstrates a gradual decline with the increase in fiber content. For a fixed fiber length of 12 mm, the 28-day strength increased from 2.62 MPa at 0.1% content to 2.97 MPa at 0.3%, then decreased to 2.81 MPa at 0.5% and further to 2.06 MPa at 0.9%. The incorporation of an optimal quantity of PVA fibers into the FLS results in the uniform dispersion of the fibers throughout the soil matrix. This configuration facilitates the establishment of a three-dimensional network in the FLS, which collectively sustains the load [44]. Additionally, the fibers exhibit notable ductility, which restricts the propagation of cracks and resists the transverse tensile stress induced by compressive deformation. However, as the fiber content continues to increase, the distribution of fibers becomes increasingly challenging due to their tendency to agglomerate, introducing internal defects that reduce compressive strength [31].

Once the content of fiber has been established, it is observed that specimens treated with 0.1% fiber exhibited an increase in strength with an increase in fiber length. Specimens treated with 0.3% and 0.5% fiber exhibited an initial increase in strength, followed by a subsequent decrease in strength with an increase in fiber length. In contrast, specimens treated with 0.7% and 0.9% fiber demonstrated a gradual decrease in strength with an increase in fiber length. At the optimal fiber content of 0.3%, the 28-day strength increased from 2.42 MPa at 3 mm to 2.53 MPa at 6 mm, 2.61 MPa at 9 mm, and reached a maximum of 2.97 MPa at 12 mm, then decreased to 2.58 MPa at 15 mm. When the fiber content is low, longer fibers enhance interlocking and form a three-dimensional staggered support network that impedes crack formation. As fiber content increases, the likelihood of fiber agglomeration rises, which reduces specimen density and impairs compressive properties [45]. Notably, as fiber length increases, the optimal fiber content tends to decrease; similarly, as fiber content increases, the optimal fiber length also decreases.

(2) Group W

**Figure 15:**
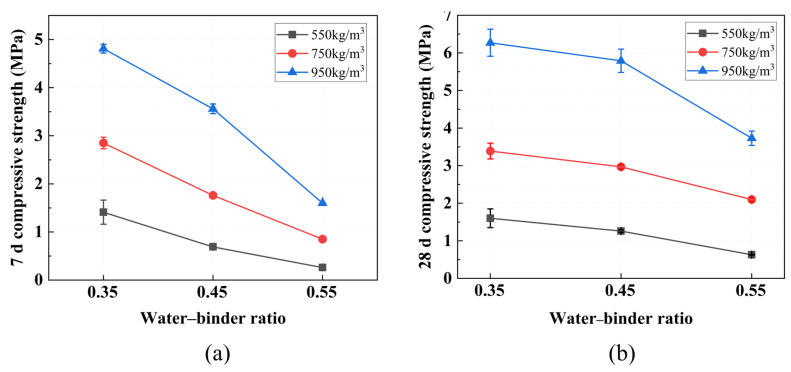
Compressive strength at different design wet densities and water–binder ratios: (**a**) 7 d; (**b**) 28 d.

The effect of varying wet densities and water–binder ratios on compressive strength is shown in Figure 15. The 28-day compressive strength ranged from 0.63 MPa (at a wet density of 550 kg/m^3^ and a water–binder ratio of 0.55) to 6.27 MPa (at 950 kg/m^3^ and 0.35), demonstrating the significant influence of these mix parameters. With the exception of the W550-0.55 mix, all specimens exceeded 1 MPa.

Furthermore, the patterns of change in 7 d and 28 d compressive strength with wet density and water–binder ratio are almost identical. The compressive strength of FLS consistently decreases with increasing water–binder ratio and increases with an increasing wet density. For a fixed wet density of 750 kg/m^3^, raising the water–binder ratio from 0.35 to 0.55 reduced the 28-day strength from 3.39 MPa to 2.97 MPa and further to 2.10 MPa. Similar trends were observed at wet densities of 550 kg/m^3^ and 950 kg/m^3^. Conversely, at a constant water–binder ratio, increasing the wet density substantially increased the strength. At a water–binder ratio of 0.45, the 28-day strength rose from 1.26 MPa at 550 kg/m^3^ to 2.97 MPa at 750 kg/m^3^ and 5.79 MPa at 950 kg/m^3^; analogous trends were observed at ratios of 0.35 and 0.55.

An increase in the water–binder ratio raises the free water content of the slurry; the surplus water evaporates, leaving behind numerous pores that weaken the internal structure of the FLS [43]. Excess water also makes bubbles buoyant, leading to stratification and the formation of interconnected air holes, which further reduce strength. As the design wet density decreases, foam content increases, generating more pores and a looser matrix [46]. At the lowest density, the material contains abundant large and interconnected voids, making it especially vulnerable to compressive failure [26,43]. These results indicate that wet density is the dominant factor governing compressive strength, while higher water–binder ratios reduce strength by increasing porosity.

### 5.5. Micro-Morphology (SEM)

(1) Group P

**Figure 16:**
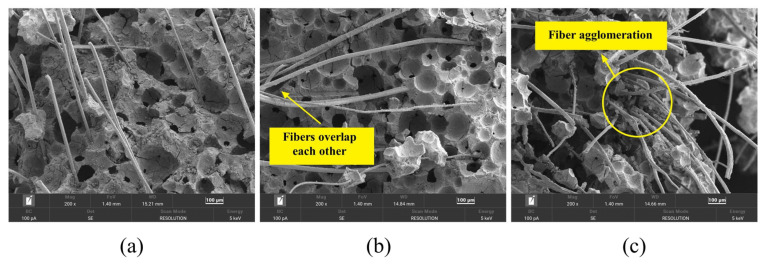
Microscopic morphology of specimens with different fiber lengths and contents: (**a**) P6-0.3; (**b**) P12-0.3; (**c**) P12-0.7.

To investigate the microstructural characteristics associated with different fiber performances, specimens representing the optimal fiber combination (P12-0.3), a lower fiber length (P6-0.3), and a higher fiber content exhibiting agglomeration (P12-0.7) were selected for SEM analysis. As shown in Figure 16, the PVA fibers are randomly distributed within the FLS and appear firmly attached to the matrix. However, the micro-morphology varies significantly with fiber length and content.

A comparison of Figure 16a,b reveals that, at the same fiber content (0.3%), the 6 mm fibers are uniformly distributed and aligned in parallel within the FLS, without forming effective lap joints with one another. This parallel distribution suggests that mechanical properties may be compromised when the loading direction aligns with the fiber orientation, which is consistent with the lower compressive strength of P6-0.3 (2.53 MPa) compared to P12-0.3 (2.97 MPa). In contrast, the 12 mm fibers are distributed more uniformly and lap with each other to form a three-dimensional mesh structure. When subjected to external forces, these interlocked fibers generate force opposite to the deformation direction, thereby resisting cracking and damage through the fiber bridging mechanism [47].

A comparison of Figure 16b,c shows that at a constant fiber length of 12 mm, significant fiber clumping occurs in the P12-0.7 specimen as the fiber content increases. Excessive fiber content leads to fiber agglomeration, which prevents uniform distribution within the slurry and introduces internal defects, consistent with previous findings that excessive fiber content leads to fiber balling and introduces internal defects [31]. The formation of fiber agglomerations reduces internal compactness and makes the specimen more susceptible to damage, as evidenced by the 17.8% decrease in compressive strength of P12-0.7 compared to P12-0.3.

(2) Group W

**Figure 17:**
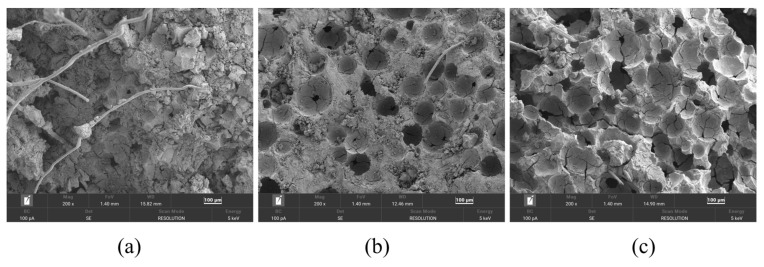
Microscopic morphology of specimens with different design wet densities and water–binder ratios: (**a**) W950-0.35; (**b**) W950-0.55; (**c**) W550-0.55.

To examine pore structure variations, specimens with extreme wet densities and water–binder ratios were selected: W950-0.35 (high density, low w/b), W950-0.55 (high density, high w/b), and W550-0.55 (low density, high w/b). As shown in Figure 17, FLS exhibits a honeycomb-like structure with numerous air voids of varying sizes, interconnected by a hardened skeleton formed by the cementitious material.

A comparison of Figure 17a,b reveals that increasing the water–binder ratio substantially increases the number of air voids in specimen W950-0.55. A substantial increase in void number and size is accompanied by more extensive interconnection and deeper penetration, as well as broken pore walls. Consequently, the structure of W950-0.55 is noticeably looser than that of W950-0.35, which is consistent with the decrease in compressive strength from 6.27 MPa for W950-0.35 to 2.10 MPa for W950-0.55, confirming that higher water–binder ratios increase porosity and weaken the matrix [43].

A comparison of Figure 17b,c shows that reducing wet density leads to a notable increase in pore number and size in specimen W550-0.55. Many voids exhibit deeper penetration, and there is a higher incidence of broken and cracked air voids. The wall skeleton becomes notably thin, and the internal architecture is highly porous. This explains why W550-0.55 exhibits the poorest compressive properties, with a 28-day strength of only 0.63 MPa, consistent with the well-established positive correlation between density and compressive strength in foamed concrete [43,48].

### 5.6. Pore Structure (MIP)

The findings of the aforementioned study suggest that variations in fiber content, fiber length, wet density, and water–binder ratio exert a substantial influence on the performance of FLS. To further examine the impact of design parameters, specifically wet density and water–binder ratio, on the pore structure of FLS at the microscopic level, three representative mixtures (W950-0.35, W950-0.55, and W550-0.55) were selected for MIP analysis at 28 days.

**Figure 18:**
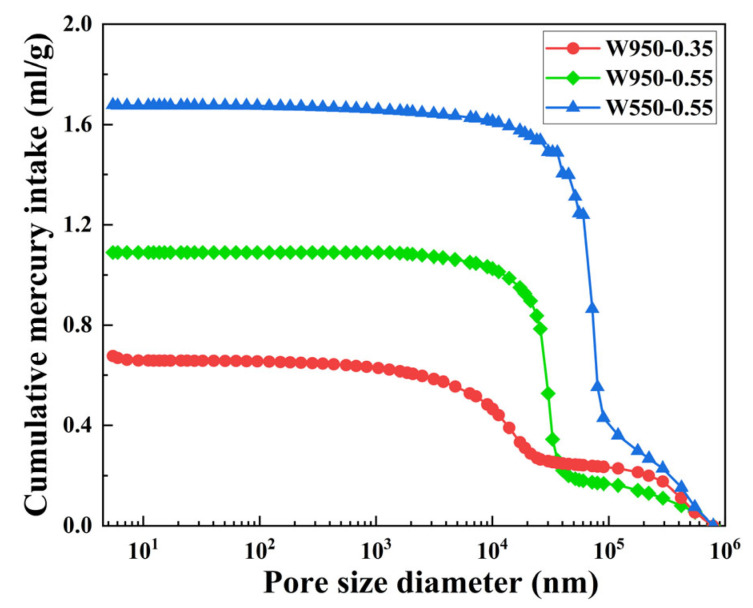
Cumulative pore volume.

The cumulative pore volume follows the order: W950-0.35 < W950-0.55 < W550-0.55, with final values of 0.66 mL/g, 1.09 mL/g, and 1.68 mL/g, respectively, as shown in Figure 18. The compressive strength follows the inverse order: W950-0.35 (6.27 MPa) > W950-0.55 (2.10 MPa) > W550-0.55 (0.63 MPa). Increasing the water–binder ratio from 0.35 to 0.55 at a constant wet density of 950 kg/m^3^ increased the cumulative pore volume by approximately 65%. Conversely, reducing the wet density from 950 to 550 kg/m^3^ at a constant water–binder ratio of 0.55 increased the cumulative pore volume by approximately 54%.

**Figure 19:**
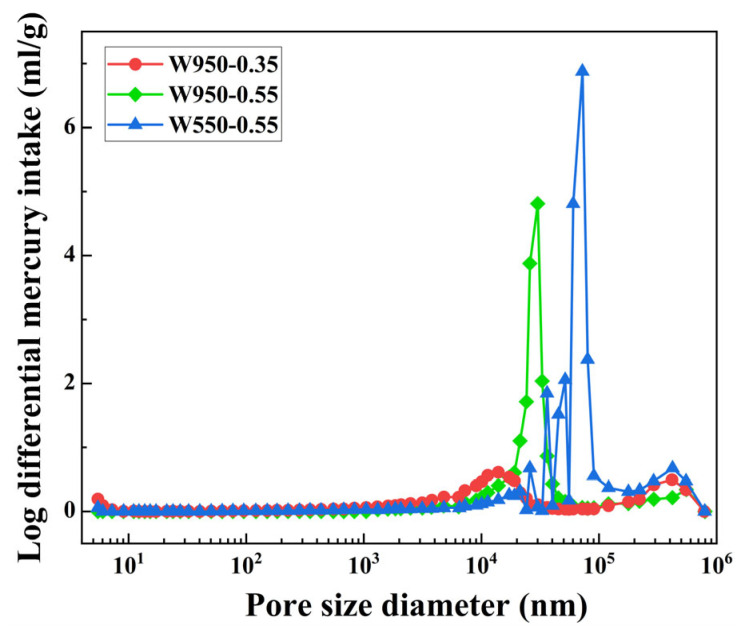
Pore size distribution.

Figure 19 illustrates the pore size distribution curves. The most probable pore diameter is approximately 30,000 nm for W950-0.55 and 72,000 nm for W550-0.55, while W950-0.35 exhibits a more diffuse distribution with no prominent single peak. Thus, the average pore size follows the same order as the most probable pore diameter: W950-0.35 < W950-0.55 < W550-0.55. Increasing the water–binder ratio increases the average pore size, while increasing wet density reduces it.

To better understand the influence of pore size on mechanical properties, pores can be classified into different categories based on their potential harmfulness following established concrete pore classification systems [48]. In these systems, pores larger than 200 nm are typically considered detrimental as they act as stress concentrators and facilitate crack propagation. For W550-0.55, pores exceeding 200 nm account for the majority of its total pore volume (approximately 1.2 mL/g out of 1.68 mL/g), while W950-0.35 contains minimal pores in this detrimental range. These large pores act as stress concentrators under compressive loading [43], resulting in the loosest internal structure and the least favorable compressive performance. In contrast, the W950-0.35 specimen exhibits a limited number of pores and a narrow pore diameter, with minimal pores in the detrimental range, resulting in a highly dense internal structure and superior compressive performance. In conclusion, the findings of the micro-pore structure analysis are consistent with those of the macro-compressive strength test.

## 6. Impact Level Analysis

To evaluate the influence of each parameter on FLS performance, analysis of variance (ANOVA) was performed on the experimental data.

(1) Group P

To evaluate the influence of fiber parameters on FLS performance, ANOVA was performed on the experimental data. The results for fiber length and fiber content are summarized in Table 6.

Table 6 presents the ANOVA results for 28-day compressive strength indicate that fiber content has a statistically significant effect on compressive strength (*p* = 0.022), whereas fiber length does not (*p* = 0.651). This suggests that, within the investigated range of 3–15 mm, fiber length plays a minor role in determining compressive strength, while fiber content is the dominant factor. The significant effect of fiber content can be attributed to the fiber bridging mechanism: a higher fiber content increases the density of the three-dimensional network within the matrix, effectively redistributing stress and inhibiting crack propagation [28,29].

For fluidity, both fiber length and fiber content show highly significant effects (*p* < 0.001). The F-value for fiber content (82.29) is larger than that for fiber length (37.21), suggesting that fiber content has a stronger influence on fluidity. A higher fiber content increases the number of fibers per unit volume, leading to more fiber–fiber and fiber–matrix interactions. This enhances internal friction and mechanical interlocking, which restricts the flow of the fresh mixture [42]. Fiber length also affects flowability, but its influence is secondary within the range tested.

(2) Group W

For mix design parameters, ANOVA was conducted to assess the effects of wet density and water–binder ratio on 28-day compressive strength and fluidity. The results are presented in Table 7.

Table 7 presents the ANOVA results for the effects of wet density and water–binder ratio on 28-day compressive strength and fluidity. Both wet density and water–binder ratio have a significant effect on compressive strength (*p* < 0.05), with wet density showing a highly significant influence (*p* = 0.001). The smaller *p*-value for wet density suggests that it contributes more to compressive strength than the water–binder ratio, which aligns with the understanding that a higher wet density produces a denser matrix with fewer pores [26]. The water–binder ratio, by contrast, has a more nuanced role; while it influences hydration, it also affects porosity, and its net effect on strength depends on finding an optimal balance.

For fluidity, both factors are highly significant (*p* < 0.001). A higher water–binder ratio increases the amount of free water and reduces internal friction between particles, thereby improving flowability. Conversely, a higher wet density increases the solid content of the mixture, which tends to restrict flow. The extremely low *p*-values for both factors confirm that they are the primary parameters governing the rheological behavior of fresh FLS, and these two factors need to be carefully balanced in practice to achieve the desired workability without compromising strength.

## 7. Constitutive Model

A parametric constitutive model is developed to describe the compressive behavior of PVA-SWFLS based on the experimental results of Group W. The experimental program was conducted in two stages: Group P (fiber length 3–15 mm, fiber content 0.1–0.9 vol%) was used to identify the optimal fiber parameters, which were found to be a fiber length of 12 mm and a fiber content of 0.3%. All subsequent constitutive modeling was performed under these fixed optimal fiber conditions using the data from Group W (water–binder ratio 0.35–0.55, wet density 550 to 950 kg/m^3^).

**Figure 20:**
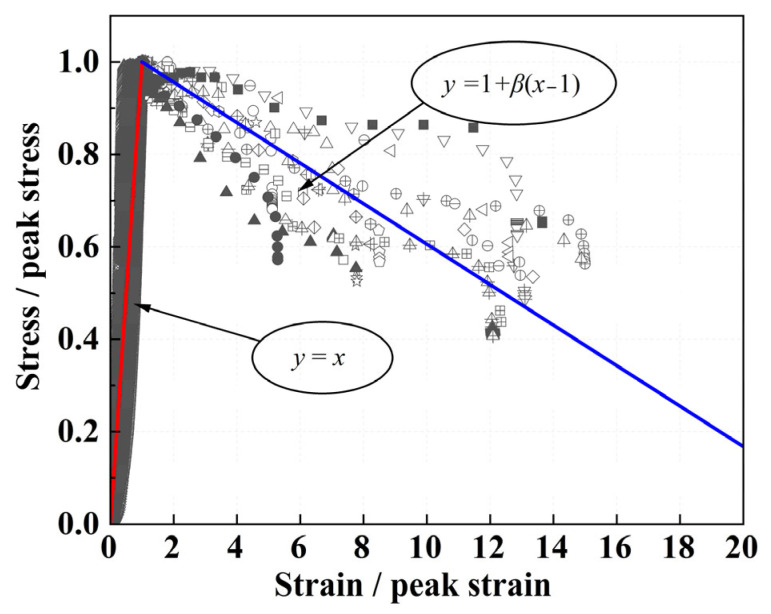
Stress–strain curves of PVA-SWFLS: model prediction for the reference mix (w = 0.45, ρ = 750 kg/m^3^) and experimental data from all 27 individual specimens.

To obtain dimensionless stress–strain curves, each experimental curve was normalized by its peak stress $\sigma_{c}$ and peak strain $\varepsilon_{c}$ using Equations (1) and (2), as shown in Figure 20. By analyzing the trends in the experimental curves, a simplified bilinear model was therefore adopted to represent the normalized stress–strain behavior, as given by Equation (3). Owing to the test setup, loading was terminated once a preset displacement was reached; the resulting vertical drop in the descending branch is an artifact of the testing procedure and was excluded from the fitting process to avoid biasing the model.

(1) $$x=\sigma /\sigma_{c}$$

(2) $$y=\varepsilon /\varepsilon_{c}$$

where σ, $\sigma_{c}$ is the actual stress at each point and the peak stress, respectively, and ε, $\varepsilon_{c}$ is the actual strain at each point and the peak strain, respectively.

(3) $$y=\left\{\begin{array}{l} x,\;\;\;\;0\leq x<1\\ 1+\beta (x-1),x\geq 1 \end{array}\right.$$

where β is the normalized descending slope, related to the ascending slope by $\beta =k/(\sigma_{c}/\varepsilon_{c})$, where k is the actual descending slope.

To establish a generalized model applicable to different mix proportions, a reference mix with water–binder ratio of 0.45 and wet density of 750 kg/m^3^ was selected. The characteristic parameters of the reference mix were determined experimentally as the average of three replicate specimens: a peak stress of 3.035 MPa, a peak strain of 0.019 (i.e., 1.9%), and a post-peak slope of k=−14.18 MPa.

Figure 20 shows the predicted stress–strain curve (solid line) for the reference mix (w = 0.45, ρ = 750 kg/m^3^), together with the measured data points from all 27 individual specimens of Group W. Black solid markers with different shapes represent the three parallel specimens of this reference mix; hollow markers of various shapes denote the remaining 24 specimens from the other seven mix proportions. It can be seen that the predicted curve captures the general trend of the experimental data.

For any other mix, the three parameters (*σ_c_*, *ε_c_* and *k*) are expressed as ratios relative to these reference values. Multiple regression analysis was performed to establish the relationships between these normalized parameters and the mix design variables. For brevity, let w denote the water–binder ratio and ρ denote the wet density (in kg/m^3^).

For peak stress, the regression yielded:

(4) $$\beta_{\sigma }=\frac{\sigma_{c}}{\sigma_{0}}=-2.809+2.839w+0.007\rho -0.007w\rho$$

with a coefficient of determination R^2^ = 0.972, indicating excellent agreement with the experimental data.

Statistical analysis showed no significant correlation between peak strain and wet density (*p* > 0.05), which is consistent with the finding of Tan et al. [49] that peak strain has no clear dependence on density in foamed concrete. Therefore, only water–binder ratio was considered, giving:

(5) $$\beta_{\varepsilon }=\frac{\varepsilon_{c}}{\varepsilon_{0}}=2.251w$$

with R^2^ = 0.875 and the slope term highly significant (*p* < 0.001).

For the post-peak slope, which is influenced by both water–binder ratio and wet density with a clear coupling effect, the regression result is:

(6) $$\beta =\frac{k}{\left(\sigma_{c}/\varepsilon_{c}\right)}=\frac{\beta_{\varepsilon }}{\beta_{\sigma }}(0.251-0.346w-0.001\rho +0.001w\rho )$$

The model yields R^2^ = 0.863 and is statistically significant overall (*p* = 0.0135).

Equations (4)–(6) are valid within the investigated ranges: w = 0.35–0.55 and ρ = 550–950 kg/m^3^. In design or analysis, for a given mix with specified w and ρ, the actual parameters $\sigma_{c}$, $\varepsilon_{c}$ and k are obtained by multiplying by the corresponding ratios from Equations (4)–(6), where $\sigma_{0}$, $\varepsilon_{0}$ could be obtained through testing. Substituting these into the bilinear model (Equations (1)–(3)) yields the full stress–strain curve.

**Figure 21:**
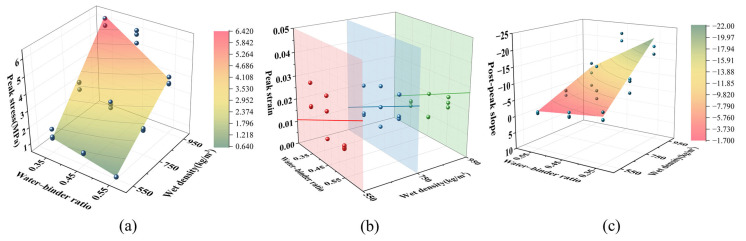
Coupled effects of water–binder ratio and density on key mechanical parameters of PVA-SWFLS: (**a**) Peak Stress; (**b**) peak Strain; (**c**) post-peak slope.

Figure 21 presents these comparisons for peak stress, peak strain, and post-peak slope, respectively, illustrating the overall agreement between the fitted curves and the measured data.

## 8. Conclusions

In this paper, a novel PVA fiber-reinforced solid waste-based foamed lightweight soil (PVA-SWFLS) was prepared using a binder system composed of RM, GGBS, and FA as a complete substitute for Portland cement. The effects of fiber content (0–0.9 vol%), fiber length (3–15 mm), water–binder ratio (0.35–0.55), and wet density (550–950 kg/m^3^) on material performance were systematically investigated. The following conclusions can be drawn:

(1) Fluidity decreases with increasing fiber content and fiber length, while it increases with higher water–binder ratio and wet density. Quantitatively, increasing fiber content to 0.9% at 15 mm length reduced fluidity by 34.9% compared to the control group.
(2) The compressive strength of PVA-SWFLS exhibits a non-monotonic dependence on fiber parameters. The optimal fiber combination is 12 mm length and 0.3% content, achieving a 28-day strength of 2.97 MPa—a 39.4% increase over the fiber-free control group. The reinforcing effect is governed by competing mechanisms: below the optimum, fibers form an effective three-dimensional network that bridges cracks and enhances strength; beyond it, fiber agglomeration introduces internal defects that degrade performance. Analysis of variance confirms that fiber content significantly affects compressive strength (*p* = 0.022), while fiber length does not (*p* = 0.651) within the tested range (3–15 mm), indicating that fiber content is the governing parameter.
(3) For mix design parameters, wet density is the dominant factor controlling compressive strength (*p* = 0.001), followed by water–binder ratio (*p* = 0.029). Strength increases consistently with higher wet density and lower water–binder ratio, with the highest strength (6.27 MPa) achieved at 950 kg/m^3^ and w/b = 0.35.
(4) Microstructural analysis reveals that compressive strength is governed not only by total porosity but critically by pore size distribution. Pores larger than 200 nm act as stress concentrators that facilitate crack propagation. For W550-0.55, pores exceeding 200 nm account for approximately 1.2 mL/g of its total 1.68 mL/g pore volume, directly explaining its poor compressive performance.
(5) A parametric constitutive model was developed for PVA-SWFLS based on W-group data, with a reference mix (w/b = 0.45, ρ = 750 kg/m^3^) serving as the baseline. The model adopts a bilinear form in which the key parameters—peak stress, peak strain, and post-peak slope—are expressed as functions of water–binder ratio and wet density. Regression analysis yielded coefficients of determination of 0.972, 0.875, and 0.863 for these three parameters, respectively.

These findings demonstrate that PVA-SWFLS is a promising sustainable construction material. For practical applications, the optimal fiber parameters (12 mm length and 0.3% content) are recommended, while wet density and water–binder ratio should be selected based on the target strength requirements of the specific engineering project, with higher wet density and lower water–binder ratio yielding greater strength.

## Figures and Tables

**Table 1:** Chemical composition of RM, GGBS, FA (%).

Composition	Fe_2_O_3_	Al_2_O_3_	SiO_2_	Na_2_O	TiO_2_	CaO	SO_3_	MgO	LOI
RM	46.1	22.2	14.0	10.3	5.4	0.7	0.6	0.2	0.7
GGBS	1.4	20.9	25.2	0.9	1.7	31.5	-	13.2	0.4
FA	7.6	33.8	49.7	0.5	1.4	2.9	1.4	0.6	2.1

**Table 2:** Properties of foaming agent.

Foam Density (kg/m^3^)	Settling Distance (mm)	Water Secretion (mL)	Bubbling Times
50	4	23	20

**Table 3:** Physical and mechanical properties of PVA fiber.

Length (mm)	Density (g/cm^3^)	Tensile Strength (MPa)	Elastic Modulus (GPa)	Fracture Elongation (%)	Diameter (μm)
3, 6, 9, 12, 15	1.31	1560	41	6.5	15

**Table 4:** Mix proportions of solid waste cementitious materials (wt.%).

Group Number	RM	GGBS	FA
A10	90	10	-
A20	80	20	-
A30	70	30	-
A40	60	40	0
A50	50	50	-
B5	55	40	5
B10	50	40	10
B15	45	40	15
B20	40	40	20

Group A testing involves the measurement and analysis of the ratio of reactive mixture (RM) to ground granulated blast furnace slag (GGBS), with the numerical values denoting the content of GGBS. Group B testing encompasses the measurement and analysis of the ratio of RM to GGBS and fly ash (FA), with the numerical values representing the content of FA.

**Table 5:** Mix proportion design of solid waste-based foamed lightweight soil.

Group		Fiber Length (mm)	Fiber Content (X) (Vol%)	Design Wet Density (kg/m^3^)	Water–Binder Ratio (Y)
CG		-	-	750	0.45
Group P	P3-X	3	0.1/0.3/0.5/0.7/0.9		
	P6-X	6			
	P9-X	9			
	P12-X	12			
	P15-X	15			
Group W	W550-Y	12	0.3	550	0.35/0.45/0.55
	W750-Y			750	
	W950-Y			950	

Group P was used to investigate the effects of different fiber contents and fiber lengths; Group W was used to investigate the effects of different wet densities and water–binder ratios. X represents different fiber contents; Y represents different water–binder ratios.

**Table 6:** Analysis of variance of experimental results for Group P.

Variables	Source	Sum of Squared Deviations	Degree of Freedom	Mean Square	F-Value	*p*-Value	Significance
28d compressive strength	Fiber Length	0.126	4	0.031	0.625	0.651	ns
	Fiber content	0.783	4	0.196	3.89	0.022	*
	Error	0.806	16	0.05	/	/	/
Fluidity	Fiber Length	4876.56	4	1219.14	37.21	<0.001	**
	Fiber content	10,784.24	4	2696.06	82.29	<0.001	**
	Error	524.24	16	32.77	/	/	/

Significance: ns = not significant (*p* > 0.05); * = significant (*p* < 0.05); ** = highly significant (*p* < 0.01).

**Table 7:** Analysis of variance of experimental results for Group W.

Variables	Source	Sum of Squared Deviations	Degree of Freedom	Mean Square	F-Value	*p*-Value	Significance
28d Compressive Strength	Design wet density	25.524	2	12.762	59.838	0.001	**
	Water–binder ratio	4.139	2	2.07	9.703	0.029	*
	Error	0.853	4	0.213	/	/	/
Fluidity	Design wet density	468.67	2	234.333	281.2	<0.001	**
	Water–binder ratio	1302	2	651	781.2	<0.001	**
	Error	3.333	4	0.833	/	/	/

Significance: * = significant (*p* < 0.05); ** = highly significant (*p* < 0.01).

## Data Availability

The original contributions presented in this study are included in the article. Further inquiries can be directed to the corresponding author.

## References

[1] Li L., Li Y. (2022). The spatial relationship between CO_2_ emissions and economic growth in the construction industry: Based on the Tapio decoupling model and STIRPAT model. Sustainability.

[2] Xue H., Lv G., Zhang T.-A. (2025). Progress of solid waste red mud in the field of ecology and environment. Water Air Soil Pollut..

[3] Tran N.P., Nguyen T.N., Ngo T.D., Le P.K., Le T.A. (2022). Strategic progress in foam stabilisation towards high-performance foam concrete for building sustainability: A state-of-the-art review. J. Clean. Prod..

[4] Archambo M., Kawatra S. (2021). Red mud: Fundamentals and new avenues for utilization. Miner. Process. Extr. Metall. Rev..

[5] Wang J., Liu B., Wang D., Sun R. (2026). Characterization of red mud and its utilization in preparing alternative cementitious materials: A review. Clean. Mater..

[6] Li X.H., Zhang Q. (2022). Influence behavior of phosphorus slag and fly ash on the interface transition zone in concrete prepared by cement-red mud. J. Build. Eng..

[7] Ma S., Liu X., Zhang Z., Shao Y., Sun Y., Li S., Du W., Zhu L., Wang J. (2026). Synergistic utilization of bauxite residue (red mud) and multiple solid wastes for low carbon precast concrete materials: Mechanical performance, mechanism and sustainability assessment. Green Energy Resour..

[8] Ghalehnovi M., Roshan N., Hakak E., Shamsabadi E.A., de Brito J. (2019). Effect of red mud (bauxite residue) as cement replacement on the properties of self-compacting concrete incorporating various fillers. J. Clean. Prod..

[9] Zhang W., Liu X., Wang Y., Li Z., Li Y., Ren Y. (2021). Binary reaction behaviors of red mud based cementitious material: Hydration characteristics and Na^+^ utilization. J. Hazard. Mater..

[10] Danner T., Justnes H. (2020). Bauxite Residue as Supplementary Cementitious Material—Efforts to Reduce the Amount of Soluble Sodium. Nord. Concr. Res..

[11] Tang L., He Z.Y., Yang R.Q., Pei S.S., Zou M., Qin M. (2024). High temperature calcined red mud-cement mortar: Workability, mechanical properties, hydration mechanism, and microstructure. Sustain. Chem. Pharm..

[12] Lin C.J., Dai W.J., Li Z.F., Sha F. (2020). Performance and Microstructure of Alkali-Activated Red Mud-Based Grouting Materials Under Class F Fly Ash Amendment. Indian Geotech. J..

[13] Feng Y.H., Zhang Z., Gao J., Feng G.P., Qiu L., Feng D.L., Zhang X.X., Zhu X. (2021). Research status of centrifugal granulation, physical heat recovery and resource utilization of blast furnace slags. J. Anal. Appl. Pyrolysis.

[14] Tripathy S.K., Dasu J., Murthy Y.R., Kapure G., Pal A.R., Filippov L.O. (2020). Utilisation perspective on water quenched and air-cooled blast furnace slags. J. Clean. Prod..

[15] Venu M., Rao G.M., Kumar Y.A., Madduru S.R.C., Bellum R.R. (2020). Influence of alkaline ratios on strength properties of fly ash-ground granulated blast furnace slag based geopolymer mortars. IOP Conference Series: Materials Science and Engineering.

[16] Li Z., Gao M., Lei Z., Tong L., Sun J., Wang Y., Wang X., Jiang X. (2023). Ternary cementless composite based on red mud, ultra-fine fly ash, and GGBS: Synergistic utilization and geopolymerization mechanism. Case Stud. Constr. Mater..

[17] Wang X., Yang Y., Sun Z., Guan D., Zhang W., Guo R. (2026). Influence of Red Mud Substitution on Strength, Hydration process, and Microstructural in GGBS-Based UHPC. Results Eng..

[18] Yuan W., Li H., Zhang D., Pan T., Li Y., Yan Z., Chang N. (2025). Effects of Red Mud Incorporation on the High-Temperature Performance of Alkali-Activated Fly Ash. J. Environ. Chem. Eng..

[19] Wang C.-Q., Yu L., Liu Y.-Y., Wu K. (2026). Red mud-modified fly ash resource utilization in foamed concrete: Basic performance research, hydration reaction and pore structure quantitative analysis. Chem. Eng. J..

[20] Mukiza E., Zhang L.L., Liu X.M., Zhang N. (2019). Utilization of red mud in road base and subgrade materials: A review. Resour. Conserv. Recycl..

[21] Yang D., Wang P., Chen W., Liu L., Huang Y., Xiang X., Wang G., Wu J. (2025). Effects of red mud, desert sand, and ground granulated blast furnace slag on the mechanical properties and microstructure of fly ash-based geopolymer. Constr. Build. Mater..

[22] Ou X.D., Zeng Y.C., Jiang J., Lyu Z., Chen H.L., Chen G.Y. (2022). Experimental research on the properties of foamed mixture lightweight soil with red mud. Case Stud. Constr. Mater..

[23] Xiong Y.L., Zhang Z.D., Huo B.B., Zhang C., Liu C., Zhang Y.M. (2024). Uncovering the influence of red mud on foam stability and pore features in hybrid alkali-activated foamed concrete. Constr. Build. Mater..

[24] Wang Z.G., Pu L.Y., Yao Y.C., Yang J., Li L.P., Luo J.R., Zhu S.Q., Zeng Q., Ruan S.Q. (2023). Unveiling the role of reactive magnesia and red mud in CO_2_-cured aerated concrete. J. Build. Eng..

[25] Song Y.F., Dong M.H., Wang Z.G., Qian X.Q., Yan D.M., Shen S.Y., Zhang L.F., Sun G.C., Lai J.Y., Ruan S.Q. (2022). Effects of red mud on workability and mechanical properties of autoclaved aerated concrete (AAC). J. Build. Eng..

[26] Shankar A.N., Chopade S., Srinivas R., Kumar Mishra N., Eftikhaar H.K., Sethi G., Singh B. (2023). Physical and mechanical properties of foamed concrete, a literature review. Mater. Today Proc..

[27] Falliano D., Parmigiani S., Suarez-Riera D., Ferro G.A., Restuccia L. (2022). Stability, flexural behavior and compressive strength of ultra-lightweight fiber-reinforced foamed concrete with dry density lower than 100 kg/m. J. Build. Eng..

[28] Shi X.X., Ning B.K., Wang J.X., Cui T.T., Zhong M.Y. (2023). Improving flexural toughness of foamed concrete by mixing polyvinyl alcohol-polypropylene fibers: An experimental study. Constr. Build. Mater..

[29] Tang R., Wei Q.S., Zhang K., Jiang S., Shen Z.M., Zhang Y.X., Chow C.W.K. (2022). Preparation and performance analysis of recycled PET fiber reinforced recycled foamed concrete. J. Build. Eng..

[30] Hu C.-f., Li L., Li Z. (2022). Effect of fiber factor on the workability and mechanical properties of polyethylene fiber-reinforced high toughness geopolymers. Ceram. Int..

[31] Wang X., Jin Y., Huang W., Li X., Ma Q. (2023). Effect of hybrid basalt and sisal fibers on durability and mechanical properties of lightweight roadbed foam concrete. Case Stud. Constr. Mater..

[32] Shi X.X., Ning B.K., Wang J.X., Cui T.T., Zhao W.F., Li A.Q. (2024). Impact resistance of polyvinyl alcohol-polypropylene fiber toughened foamed concrete under low-velocity impact: Experiments and 3D meso-scale modelling. Case Stud. Constr. Mater..

[33] Li S., Qiu S., Zheng H., Yu H. (2026). Influence of fibers on the multiscale properties of fly ash and slag based geopolymer foam concrete. Constr. Build. Mater..

[34] Li J., Hajimohammadi A., Kim T. (2024). The surface treatment of PVA fibres to enhance fibre distribution and mechanical properties of foam concrete. Constr. Build. Mater..

[35] (2012). Methods for Testing Uniformity of Concrete Admixture.

[36] (2021). Test Method of Cement Mortar Strength (ISO Method).

[37] (2012). Technical Specification for Foamed Mixture Lightweight Soil Filling Engineering.

[38] (2008). Test Methods of Autoclaved Aerated Concrete.

[39] Arularasi V., Thamilselvi P., Avudaiappan S., Saavedra Flores E.I., Amran M., Fediuk R., Vatin N., Karelina M. (2021). Rheological behavior and strength characteristics of cement paste and mortar with fly ash and GGBS admixtures. Sustainability.

[40] Alam S., Das S.K., Rao B.H. (2017). Characterization of coarse fraction of red mud as a civil engineering construction material. J. Clean. Prod..

[41] Zhang G., Zhang P., Guo J., Hu S. (2024). Effects of PVA fibers and nano-SiO_2_ on rheological properties of geopolymer mortar. Nanotechnol. Rev..

[42] Dhasindrakrishna K., Pasupathy K., Ramakrishnan S., Sanjayan J. (2022). Rheology and elevated temperature performance of geopolymer foam concrete with varying PVA fibre dosage. Mater. Lett..

[43] Jierula A., Li H., Chen Y., Wu C., Wu X., Yin H. (2024). Study on the influence of density and water–cement ratio on the cement utilization, fluidity, mechanical properties, and water absorption of foam concrete. Buildings.

[44] Li J., Hajimohammadi A., Yu Y., Lee B.Y., Kim T. (2023). Mechanism of PVA fiber influence in foam concrete: From macroscopic to microscopic view. J. Mater. Civ. Eng..

[45] Chen L., Li P., Guo W., Zhang D., Wang R., Gao M., Yuan K. (2025). Mechanical properties and failure modes of polypropylene fiber-reinforced foamed concrete subjected to high strain rates and large compressive deformation: Effects of fiber content and length. J. Sustain. Cem.-Based Mater..

[46] Gencel O., Subasi S., Ustaoglu A., Sari A., Marasli M., Hekimoglu G., Kam E. (2022). Development, characterization and thermo-regulative performance of microencapsulated phase change material included-glass fiber reinforced foam concrete as novel thermal energy effective-building material. Energy.

[47] Jiang J., Chen M., Yu X., Jin Y., Jiang W. (2026). Experimental and microstructural investigation on the strength and frost resistance of basalt fiber reinforced steel slag foamed concrete. Sci. Rep..

[48] Gencel O., Bilir T., Bademler Z., Ozbakkaloglu T. (2022). A detailed review on foam concrete composites: Ingredients, properties, and microstructure. Appl. Sci..

[49] Tan X., Chen W., Liu H., Chan A.H.C. (2018). Stress-strain characteristics of foamed concrete subjected to large deformation under uniaxial and triaxial compressive loading. J. Mater. Civ. Eng..

